# Dynamics of the Enteric Virome in a Swine Herd Affected by Non-PCV2/PRRSV Postweaning Wasting Syndrome

**DOI:** 10.3390/v13122538

**Published:** 2021-12-17

**Authors:** Alba Folgueiras-González, Robin van den Braak, Martin Deijs, Wikke Kuller, Steven Sietsma, Valentijn Thuring, Lia van der Hoek, Ad de Groof

**Affiliations:** 1 Department Discovery & Technology, MSD Animal Health, Wim de Körverstraat 35, P.O. Box 31, 5830 AA Boxmeer, The Netherlands; alba.folgueiras.gonzalez@merck.com (A.F.-G.); robin.braak.van.den@merck.com (R.v.d.B.); 2 Laboratory of Experimental Virology, Department of Medical Microbiology, Amsterdam UMC Location AMC, University of Amsterdam, Meibergdreef 9, 1105 AZ Amsterdam, The Netherlands; m.deijs@amsterdamumc.nl (M.D.); c.m.vanderhoek@amsterdamumc.nl (L.v.d.H.); 3 ULP-Farm Animal Practice, Reijerscopse Overgang 1, 3481 LZ Harmelen, The Netherlands; wkuller@ulp.nu (W.K.); ssietsma@ulp.nu (S.S.); 4 Pig Veterinary Practice Varkensartsen Nederland, Markgraven Dierenartsen, Almeloseweg 169, 7615 NA Harbrinkhoek, The Netherlands; valentijn@varkensartsen.nl

**Keywords:** swine, postweaning, wasting, failure to thrive syndrome (PFTS), virome, metagenomics, rotavirus, astrovirus, sapovirus, enterovirus G, VIDISCA-NGS, PCV2, PRRSV

## Abstract

A commercial pig farm with no history of porcine circovirus 2 (PCV2) or porcine reproductive and respiratory syndrome virus (PRRSV) repeatedly reported a significant reduction in body weight gain and wasting symptoms in approximately 20–30% of the pigs in the period between three and six weeks after weaning. As standard clinical interventions failed to tackle symptomatology, viral metagenomics were used to describe and monitor the enteric virome at birth, 3 weeks, 4 weeks, 6 weeks, and 9 weeks of age. The latter four sampling points were 7 days, 3 weeks, and 6 weeks post weaning, respectively. Fourteen distinct enteric viruses were identified within the herd, which all have previously been linked to enteric diseases. Here we show that wasting is associated with alterations in the enteric virome of the pigs, characterized by: (1) the presence of enterovirus G at 3 weeks of age, followed by a higher prevalence of the virus in wasting pigs at 6 weeks after weaning; (2) rotaviruses at 3 weeks of age; and (3) porcine sapovirus one week after weaning. However, the data do not provide a causal link between specific viral infections and the postweaning clinical problems on the farm. Together, our results offer evidence that disturbances in the enteric virome at the preweaning stage and early after weaning have a determining role in the development of intestinal barrier dysfunctions and nutrient uptake in the postweaning growth phase. Moreover, we show that the enteric viral load sharply increases in the week after weaning in both healthy and wasting pigs. This study is also the first to report the dynamics and co-infection of porcine rotavirus species and porcine astrovirus genetic lineages during the first 9 weeks of the life of domestic pigs.

## 1. Introduction

The postweaning period is a critical stage in the life of pigs, with sudden social, environmental, and immunological changes. This is sometimes associated with clinical disease problems generally referred to as ‘wasting disease’. Pigs appear healthy at weaning, at desired body weights and with sufficient solid feed intake, but during the subsequent 2–3 weeks the pigs’ body condition deteriorates with a clear lack of growth and loss of finisher-type musculature. In extreme cases, pigs may even die or may have to be euthanized due to cachexia—i.e., pathological physical wasting typified by a loss of weight and muscle mass, involving lipolysis, activation of the acute phase response and insulin resistance [1]. It was regarded as an occasional problem in swine husbandry for many years and was typically overlooked as it entailed marginal economic loses. The scenario drastically changed between 2008 and 2010, however, when several farms in North America reported a significant increase in the number and frequency of wasting disease cases [2,3]. The condition was named porcine peri-weaning failure to thrive syndrome (PFTS) and was later also reported in Spain and Brazil [4,5], though the condition is likely present worldwide but is either neglected or named differently (wasting disease in most cases) due to its multifactorial symptomatology, lack of distinct clinical signs and specific pathological lesions, and as yet unknown origin. Morbidity can range between 1% and 20% on affected farms, with an increase in reported mortalities up to half of that percentage, being highly variable between farms and among different litters from the same herd [2,3,6].

Due to the complexity described above, differential diagnosis has been difficult and there is still no agreement in the field about whether the so-called failure to thrive or wasting is caused by infectious agents, genetic predisposition, environmental factors, nutritional factors, or a combination of these. The clinical presentation shows similarities to postweaning multisystemic wasting syndrome (PMWS)—a multifactorial disease that causes a rapid weight loss in weaned piglets—with the difference that the above-mentioned wasting cases are not correlated with the presence of porcine circovirus type 2 (PCV2) [1,3]. The clinical presentation has also been confused with porcine reproductive and respiratory virus (PRRSV) symptomatology, but the absence of this virus in affected herds again suggests a different and possibly multifactorial etiology [3].

It has become evident during the last few years that Koch’s one pathogen–one disease hypothesis often fails to explain clinical disease problems that occur in farm animal populations. Multiple microorganisms, including viruses, may co-infect the host, creating a specific clinical symptomatology that is difficult to dissect because multiple pathogens are present. Furthermore, multifactorial disease often arises due to a disbalance in several factors, including infectious agents, with a clinical appearance that is diverse and therefore not easily dissectible.

It is essential that, when investigating the etiology of a multifactorial disease, not only individual viruses but the entire virome is characterized. Temporal changes in the virome may be important, as it is expected that pathogenic viruses show a viremic peak prior to the onset of clinical or pathological changes, followed by a reduction in viral load. Viral metagenomics using unbiased next generation sequencing is a suitable technology to identify such virome changes, allowing us to increase our knowledge of virosphere diversity and providing us with a tool to elucidate the etiology of diseases the cause of which is yet unknown [7,8]. One of these unbiased virus discovery methods is VIDISCA, virus discovery cDNA-AFLP (amplified fragment length polymorphism) [9], which has been successfully used to detect a large number of novel human and animal viruses in cell culture supernatants [10], sera [11,12], feces [11,12], respiratory clinical samples [9], cerebrospinal fluid [13,14], and multiple solid tissues [15].

In the present study, we used viral metagenomics to follow pigs from birth till about 12 weeks of age on a farm with a long-term history of wasting pigs after weaning (up to 20–30% of postweaning pigs). We characterized the viral communities in rectal swabs before, during, and after the onset of clinical wasting symptoms. A longitudinal sampling strategy was chosen to identify viral agents and to compare viral loads at different ages in healthy and clinically affected pigs with the aim of identifying possible viral agents connected with wasting symptomology. The dynamics of two prominent viral families circulating in the farm, rotaviruses and astroviruses, were subsequently studied in more detail.

Rotaviruses, belonging to the Reoviridae family, are one of the main causes of severe neonatal diarrhea in animals and humans. They are non-enveloped, 11-segmented, double-stranded RNA viruses [16]. Based on the sequencing of the VP6 protein, rotaviruses have been classified into eight antigenically distinct species (A–J), five of which have been identified in pigs (RVA, RVB, RVC, RVE, and RVH) [16,17]. RVA was the first species to be identified in pigs and it is considered the most prevalent and pathogenic RV worldwide. Although primarily solely associated with neonatal diarrhea, co-infections with other enteric infectious agents are commonly reported [18]. In contrast, porcine RVB, RVC, and RVH have only sporadically been associated with diarrhea outbreaks in suckling piglets and are often found together with RVA [19,20,21]. Nonetheless, porcine RVB has recently been described as a primary causative agent of diarrhea outbreaks in newborn pigs in Brazil [22]. Albeit the role of rotaviruses in neonatal diarrhea is well accepted, less is known about their role after weaning.

Porcine astrovirus, is a non-enveloped, single-stranded, positive-sense RNA virus belonging to the family Astroviridae. First characterized in piglets suffering from diarrhea in 1980 [23], the virus has since then been identified worldwide and further divided into five distinct genetic lineages (PAstV1–5). Porcine astroviruses are highly prevalent in feces of diarrheic but also healthy piglets, and its potential association with clinical disease may have been overlooked [24,25,26]. Recently, porcine astroviruses have gained attention due to the appearance of new reports linking certain genotypes with disease. Several independent studies have identified PAstV3 in the central nervous system (CNS) of newly weaned pigs with encephalomyelitis and posterior paraplegia of unknown origin [27,28,29]. PAstV5 has been described in association with enteritis in weaned pigs [30,31]. Moreover, PAstV4 has been correlated with neonatal diarrhea and also found in high levels in nasal swabs from pigs with unexplained respiratory disease [31,32]. Overall, porcine astroviruses appear in combination with other enteric infections—e.g., porcine rotavirus A and C, porcine kobuvirus, porcine circovirus 2, sapovirus, transmissible gastroenteritis virus, porcine epidemic diarrhea virus [33,34]—which suggests its possible role in a multifactorial causation of disease.

We present the first longitudinal study on enteric viral dynamics in a herd affected by postweaning wasting complications that are not related to PCV2 or PRRSV infections. We provide insight into the virus families that play a role in the characteristic symptomatology in the herd and their dynamics.

## 2. Materials and Methods

### 2.1. Farm Description and Diagnostic Sampling Strategy

A commercial growing phase swine production farm located in the Netherlands with a long (>2 years) history of wasting disease was selected for the study. There was no history of porcine circovirus 2 (PCV2)/porcine reproductive and respiratory syndrome virus (PRRSV) on this particular farm.

Pigs were reported to start wasting 2 weeks after weaning with characteristic symptoms, such as long hair, bad body condition score, reduced feed and water intake, and the notable absence of typical musculature development that is typical for the swine breed TN70 x TN Tempo (Topigs Norsvin, Helvoirt, The Netherlands). Sometimes, pigs’ discomfort was such that they showed tail and ear biting behavior. Five to fifteen percent of the animals were affected and mortality was above 4%. No clear diagnosis was acquired from pathology, blood, and feces sampling other than “intestinal disorder”. Water pipes were cleaned and different feeding strategies were applied without result. In addition, several vaccinations (*Mycoplasma hyopneumoniae*, porcine circovirus type 2 (PCV2), *Lawsonia intracellularis*, Shiga toxin, F4- and F18-positive enterotoxigenic *E. coli* strains) and antibiotic treatments (paromycine, oxytetracycline, colistine, tulathromycine) were applied in weanling piglets. However, after 2 years, no clinical improvement was achieved, despite many interventions.

**Figure 1:**
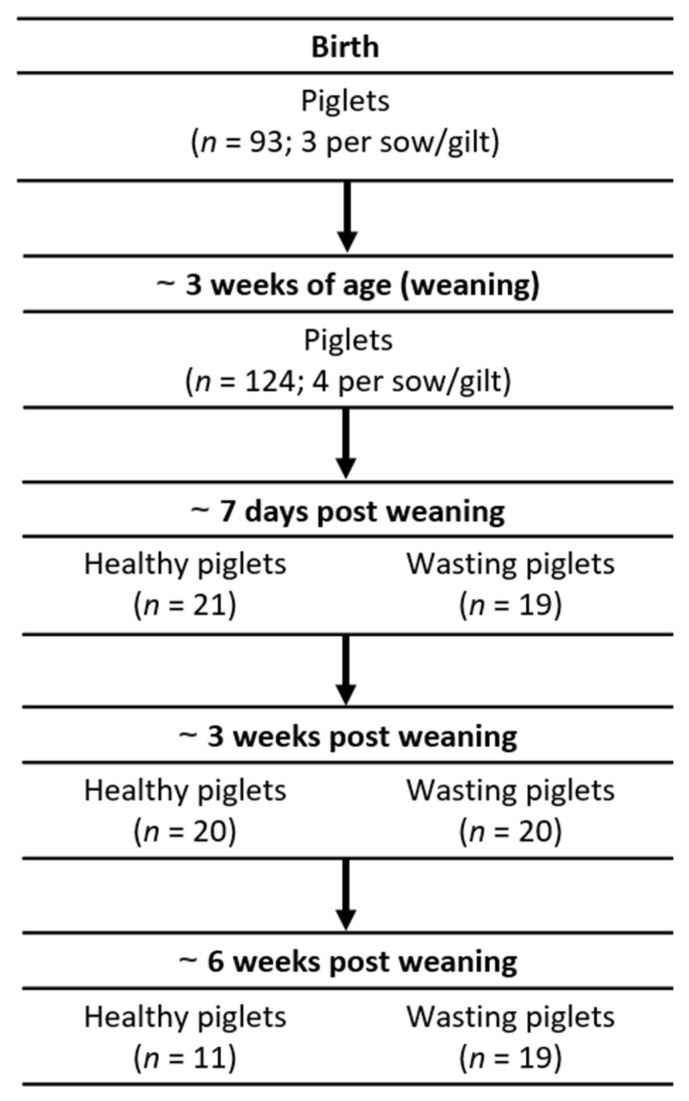
Sampling strategy showing sampling time points and number of animals sampled (total number of samples collected *n* = 327).

For this longitudinal study, a rectal swab sampling strategy at multiple time points was initiated to collect materials for metagenomics analysis, as illustrated in Figure 1. A total of 31 litters (seven from gilts, 24 from sows) from two consecutive farrowing rounds were included in this study (total number of pigs followed *n* = 377). Rectal swabs were collected at birth from three randomly selected piglets from each litter. At three weeks of age (just before weaning at approximately day 25), four piglets per litter were randomly selected for rectal swab sampling. Thereafter, rectal swab sampling was performed at approximately 7 days, 3, and 6 weeks post weaning. Wasting pigs (score 2, score 3, see below) were sampled along with a random selection of clinically healthy-appearing animals (score 1) as controls. All individual animals were clinically scored and selection for sampling was carried out randomly without reference to the previous time point or points. There was no design made to repeatedly sample individual pigs. For most pigs sampled at the latest two time points we could, however, trace back rectal swabs taken and allocate them to the groups as an additional analysis parameter.

Clinical scores were based on visual appearance (1—healthy appearance; 2—observable lower weight combined with loss of typical finisher-type musculature, mild to moderate wasting; 3—severely reduced weight and extensive loss of musculature, long hair, bad body condition, severe wasting). The scores were assigned to each of the piglets (*n* = 377) by a veterinarian at all the sampling points after weaning. The weight of the pigs at 3 and 6 weeks post weaning was measured using a household scale. The individual weights were determined from pigs with score 2 (*n* = 4, 3 wpw; *n* = 44, 6 wpw) and score 3 (*n* = 16, 3 wpw; *n* = 7, 6 wpw), and from a representative number of randomly chosen score 1 pigs (*n* = 20, 3 wpw; *n* = 34, 6 wpw).

### 2.2. Sample Collection

Rectal samples were collected using swabs (MWE, Corsham, UK) and immediately stored in 1 mL Sigma Virocult vials (MWE, Corsham, UK). Samples were transported to the laboratory at ambient temperature and stored at 2–8 °C until processed on the same day. In brief, samples were vortexed, transferred to 1.5 mL Eppendorf tubes, and centrifuged for 10 min at 10,000× *g* at 4 °C. Supernatants of the samples were subsequently stored at −80 °C until further processing.

Metagenomic analysis was performed on a limited number of randomly selected samples, considering sample availability and metagenomic sequencing capacity, from each age group and clinical condition, as shown in Table 1.

### 2.3. Vidisca-NGS

VIDISCA-NGS analysis was performed as described by de Vries et al. [9]. In short, 110 μL processed rectal swab suspension sample in Virocult (MWE, Corsham, UK) was centrifuged at 5000× *g* for 10 min and the supernatant was treated with TURBO™ DNase (Thermo Fisher Scientific, Waltham, MA, USA). Thereafter, nucleic acids were isolated by the Boom method [35], followed by reverse transcription with non-ribosomal random hexanucleotides [36] and second DNA strand synthesis with Klenow polymerase (New England Biolabs, Ipswich, MA, USA). DNA was digested with MseI (T^TAA; New England Biolabs, Ipswich, MA, USA) and ligated to adaptors incorporating a sample identifier sequence. DNA fragments with lengths between 100 and 400 bp were then purified by size selection with AMPure XP beads (Beckman Coulter, Brea, CA, USA). Next, a 28-cycle PCR with adaptor-annealing primers was performed on the purified fragments and DNA concentration and fragment length of the libraries was assessed using Quant-it dsDNA HS Qubit kit (Thermo Fisher Scientific Waltham, MA, USA) and the Bioanalyzer (High Sensitivity Kit, Agilent Genomics, Santa Clara, CA, USA), respectively. Seventy sample libraries were pooled at an equimolar concentration [9,12,13]. Sequencing was performed on the Ion Torrent PGM™ platform or the S5 platform (Thermo Fisher Scientific, Waltham, MA, USA) using the ION 316 Chip (PGM™ platform) and the ION 510 Chip (S5 platform) with 400 bp read length and 2 million sequences per run.

To identify and classify viral reads and background, the obtained reads were translated into protein sequences and then aligned to the NCBI eukaryotic viral Identical Protein groups using UBLAST and analyzed with the VIDISCA bioinformatics workflow [37] and an online metagenomic profiler (Taxonomer) [38]. Confirmation as a viral read was proved when the initial VIDISCA read could be aligned at the nucleotide level to a virus reference sequence with an identity over 80% using CodonCode Aligner (version 6.0.2). The number of viral reads per sample was established as the sum of original VIDISCA reads aligned to a virus reference sequence.

### 2.4. Rotavirus A and Rotavirus C Detection in Clinical Samples by Multiplex RT-qPCR

RNA was extracted for RT-qPCR from 200 µL processed rectal swab suspension sample in Virocult (MWE, Corsham, UK) by the automated MagNA Pure 96 system (Roche Applied Science, Manheim, Germany) using the protocol ‘Viral NA plasma external lysis SV3.1′. Rotavirus A (RVA) and Rotavirus C (RVC) RT-qPCR multiplex assay was performed as described by Marthaler et al. [19]. Primers and hydrolysis probes were designed on RVA and RVC VP6 sequences. The Ambion Path-ID™ Multiplex One-Step RT-PCR Kit^®^ (Life Technologies, Austin, TX, USA) was used for the RVAC RT-qPCR assay as follows. Reactions were performed in a final volume of 25 µL containing 12.5 µL 2× Multiplex RT-PCR Buffer, 1 µL Multiplex RT-PCR Enzyme Mix, 0.375 µL RVA forward primer, 0.375 µL RVA reverse primer, 2 µL RVA probe 1, 1 µL RVA probe 2, 0.375 µL RVC forward primer, 0.375 µL RVC reverse primer, 2 µL RVC, and 5 µL of the RNA isolate. Primer and probe sequences are shown in Supplementary Table S1. Thermocycling was performed in the CFX96 Touch real-time PCR detection system (Bio-Rad Laboratories, Hercules, CA, USA) starting with the reverse transcription reaction for 10 min at 45 °C, followed by the Taq activation for 10 min at 95 °C and 40 cycles of 15 s at 95 °C (denaturation) and 45 s at 60 °C (annealing and elongation). Results were analyzed with the CFX Manager software (Bio-Rad Laboratories, Hercules, CA, USA).

### 2.5. Rotavirus B Detection in Clinical Samples by RT-qPCR

A Rotavirus B (RVB) qPCR assay was designed based on RVB VP2 sequences available from the metagenomic VIDISCA-NGS analysis. First, cDNA was synthesized using the QuantiTect reverse transcription kit (Qiagen, Hilden, Germany) following the manufacturers’ guidelines. Thereafter, qPCR reactions were performed in a final volume of 25 µL containing 12.5 µL SsoAdvanced Universal SYBR Green Supermix (Bio-Rad Laboratories, Hercules, CA, USA), 6.5 µL water, 0.5 µL RVB forward primer, 0.5 µL RVB reverse primer, and 5 µL of the cDNA sample. Primer sequences are shown in Supplementary Table S1. Thermocycling was performed in the CFX96 Touch real-time PCR detection system (Bio-Rad Laboratories, Hercules, CA, USA) starting with a pre-denaturation step of 3 min at 95 °C, followed by 40 cycles of 5 s at 95 °C (denaturation) and 10 s at 59 °C (annealing and elongation). Melting curve analysis between 65 °C and 95 °C with an increasing gradient of 0.5 °C/s was performed to validate the specificity of the qPCR. Results were analyzed with the CFX Manager software (Bio-Rad Laboratories, Hercules, CA, USA).

### 2.6. Rotavirus H Detection in Clinical Samples by RT-qPCR

A Rotavirus H (RVH) RT-qPCR assay was designed based on RVH VP2 sequences available from the metagenomic VIDISCA-NGS analysis. Reaction mix and thermocycling were performed as described above for RVB using RVH forward primer and RVH reverse primer. Primer sequences are shown in Supplementary Table S1.

### 2.7. Astrovirus Detection in Clinical Samples by Multiplex RT-qPCR

Two multiplex RT-qPCR assays, one for the detection of Porcine Astrovirus 1 (PAstV1) and 2 (PAstV2) and one for the detection of Porcine Astrovirus 3 (PastV3), 4 (PAstV4), and 5 (PAstV5), were adapted from Xiao et al. [26]. The Ambion Path-ID™ Multiplex One-Step RT-PCR Kit^®^ (Life Technologies, Austin, TX, USA) was used for performing the assays.

The PAstV1-2 RT-qPCR reactions were performed in a final volume of 25 µL containing 12.5 µL 2× Multiplex RT-PCR Buffer, 1 µL Multiplex RT-PCR Enzyme Mix, 4.5 µL water, 0.5 µL PAstV forward primer, 0.5 µL PAstV reverse primer, 0.5 µL PAstV1 probe, 0.5 µL PAstV2 probe and 5 µL of the RNA isolate. Primer and probe sequences are shown in Supplementary Table S1. Thermocycling was performed in the CFX96 Touch real-time PCR detection system (Bio-Rad Laboratories, Hercules, CA, USA) starting with the reverse transcription reaction for 10 min at 45 °C, followed by the Taq activation for 10 min at 95 °C and 40 cycles of 15 s at 95 °C (denaturation) and 30 s at 55 °C (annealing and elongation). Results were analyzed with the CFX Manager software (Bio-Rad Laboratories, Hercules, CA, USA).

The PAstV3-4-5 RT-qPCR reactions were performed in a final volume of 25 µL containing 12.5 µL 2× Multiplex RT-PCR Buffer, 1 µL Multiplex RT-PCR Enzyme Mix, 4 µL water, 0.5 µL PAstV forward primer, 0.5 µL PAstV reverse primer, 0.5 µL PAstV3 probe, 0.5 µL PAstV4 probe, 0.5 µL PAstV5 probe, and 5 µL of the RNA isolate. Primer and probe sequences are shown in Supplementary Table S1. Thermocycling was performed as described before for the PAstV1-2 RT-qPCR.

### 2.8. Statistics

Statistical analysis was conducted with GraphPad Prism 8.1.1 software (GraphPad Software, Inc., San Diego, CA, USA). MS-Excel was used to manage production parameters, VIDISCA-NGS hits, and detection frequencies in RT-qPCR data.

Group differences were assessed using analysis of variance (ANOVA). The homogeneity of the variances was assessed with Bartlett’s test. If an ANOVA test was significant (*p* < 0.05), then pairwise tests with Tukey’s adjustment were used to assess specific group differences. The results were considered significant at *p* < 0.05. The Kruskal–Wallis test was used when appropriate. In this case, multiple comparison analysis was calculated using Dunn’s test. The results were considered significant at *p* < 0.05.

Viral prevalence between age groups was assessed using the chi-square test or the Fisher-exact test as correspondent, following a two-sided approach with a 95% confidence interval. The results were considered significant when *p* < 0.05.

## 3. Results

### 3.1. Description of Wasting Cases

A total of 31 litters (seven from gilts, 24 sows) were followed in this study. No clinical abnormalities were scored during the farrowing period. Starting from day 7 post weaning (dpw), pigs were individually scored with the aim of exactly describing the clinical development of wasting.

**Figure 2:**
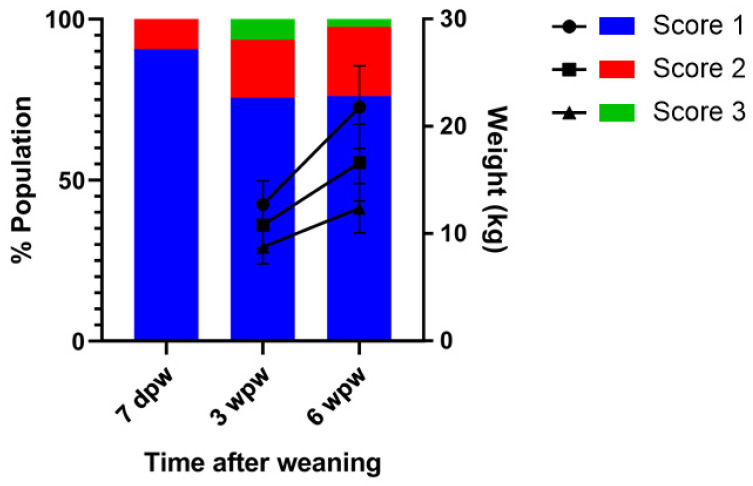
Farm performance parameters measured 7 days post weaning (7 dpw), 3 weeks post weaning (3 wpw), and 6 weeks post weaning (6 wpw). Bars represent the percentage of the pigs scored as healthy appearing (left, y-axis; score 1, blue), mild to moderate wasting (score 2, red) and severe wasting (score 3, green). Weights were determined at the last two time points and are presented as average weight and standard deviation thereof within each score group (right, y-axis).

At the time of weaning, at approximately day 25 of life, pigs were on average at the expected body weight and none of them showed any sign of wasting, albeit normal weight variation within litters was present. The clinical development of wasting started with a 9.3% (35/377) mild to moderate wasting cases (score 2) at 7 dpw, while the rest of the piglets showed a normal body condition (90.7%; 342/377; Figure 2). Two weeks later, 75.6% (285/377) of the pigs were scored healthy and on average performance (score 1), while 18.0% (68/377) scored as mildly to moderately wasting (score 2) and 6.4% (24/377) as severely wasting (score 3). At this time the body weights of the pigs in the different score groups were significantly different (one-way ANOVA, *p* < 0.0001). The group of healthy animals averaged 12.7 ± 2.2 kg, while animals with a score of 3 showed a significantly lower weight (8.7 ± 1.6 kg; Tukey’s test, *p* < 0.0001). Significant differences were not observed between the average weight in the score 2 group (10.8 ± 2.1 kg) and the other groups (Tukey’s test, group score 1 vs. group score 2: *p* = 0.19; group score 2 vs. group score 3: *p* = 0.17).

At 6 weeks post weaning, around 9–10 weeks of age, 76.1% (287/377) of the pigs scored healthy, 21.5% (81/377) showed mild to moderate wasting symptoms, and 2.4% (9/377) were diagnosed as severely wasting. The weights of the pigs in the different score groups were significantly different (one-way ANOVA, *p* < 0.0001; Figure 2). The average weight of the healthy animals (score 1; 21.8 ± 3.8 kg) was significantly higher than the average weight of the severely wasting pigs (score 3; 12.4 ± 2.3 kg; Tukey’s test, *p* < 0.001) and mild to moderately wasting pigs (score 2; 16.6 ± 3.6 kg Tukey’s test, *p* = 0.01). The pigs with mild to moderate symptoms showed a significantly higher weight than the severely wasting pigs (Tukey’s test, *p* = 0.004).

The development of clinical symptoms was similar to observations made earlier on this particular farm and also matched more generic descriptions of failure to thrive syndrome. Of note, a subset of pigs (about 10%) that scored with wasting at 3 wpw recovered back to a normal score (Table 2; light green), albeit with a noticeably lower body weight, whereas other pigs did not recover, or even worsened, and literally failed to thrive (Table 2; orange).

### 3.2. Metagenomic Analysis of the Rectal SWAB Virome

A metagenomic analysis was performed on samples collected during the full study period (see Table 1 in the Materials and Methods section).

**Figure 3:**
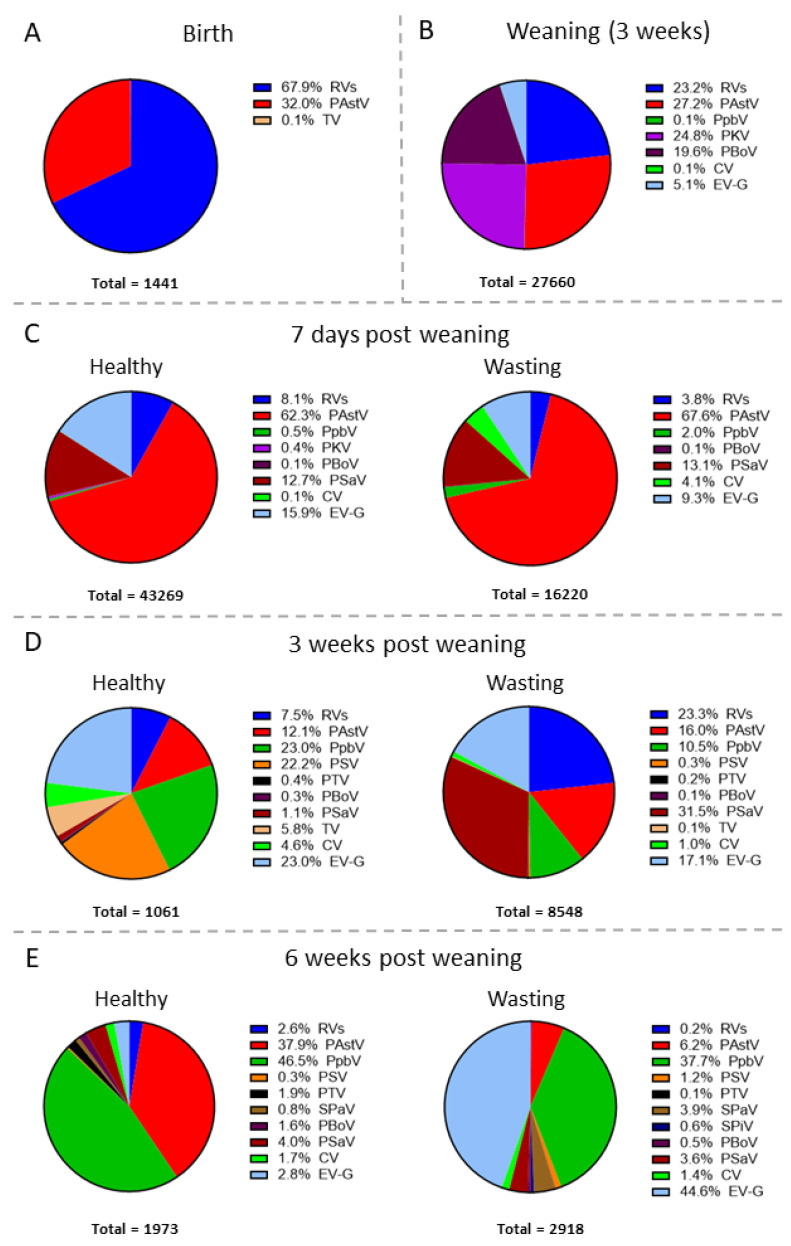
Detection of viral sequences in rectal swab samples from pigs at different ages and health status using VIDISCA-NGS.: (**A**) birth; (**B**) weaning (3 weeks); (**C**) 7 days post weaning; (**D**) 3 weeks post weaning; (**E**) 6 weeks post weaning. The legend shows the percentage of the total number of viral reads allocated to each virus. RVs: rotavirus; PAstV: porcine astrovirus; CV: circovirus; PpbV: porcine picobirnavirus; PKV: porcine kobuvirus; PSV: sapelovirus A; PTV: porcine teschovirus; SPaV: swine pasivirus; SPiV: swine picornavirus; PBoV: porcine bocavirus 3; PSaV: porcine sapovirus; TV: towan virus; EV-G: enterovirus G.

A total of 14 distinct viruses were identified in rectal swabs taken at the various time points (Figure 3). In the samples collected at birth (Figure 3A, *n* = 6), a majority of the identified sequences belonged to the genus *Rotavirus* (67.9%) and *Mamastrovirus* (Porcine Astroviruses; 32.0%). At three weeks of age (Figure 3B, *n* = 39), four viruses constituted a majority in the virome population: 27.2% of the sequences matched to porcine astroviruses, 24.8% to porcine kobuvirus (PKB), 23.2% to rotaviruses, and 19.6% to porcine bocavirus 3 (PBoV). Additionally, a small percentage of the sequences was assigned to enterovirus G (EV-G; 5.1%).

At 7 days post weaning (Figure 3C), we differentiated the virome composition of healthy (*n* = 14) and mild to moderately wasting animals (*n* = 8). In both groups, the majority of the sequences identified matched porcine astroviruses (62.3% and 67.6%, respectively). Other sequences in healthy animals matched with enterovirus G (EV-G; 15.9%), porcine sapovirus (PSaV; 12.7%) and rotaviruses (8.1%). In mild to moderately wasting pigs, 13.1% of the sequences corresponded to porcine sapovirus, followed by enterovirus G (EV-G; 9.3%), the Circovirus genus (CV; 4.1%; not PCV2/3), rotaviruses (3.8%), and porcine picobirnavirus (PpbV; 3.4%). None of the viruses showed a statistically significant difference at 7 days post weaning.

Three weeks after weaning (Figure 3D), the diversity of viruses found in the metagenomic analysis of the fecal samples increased. The majority of the sequences detected in the healthy animals (*n* = 12) corresponded to porcine picobirnavirus (PpbV; 23.0%), enterovirus G (EV-G; 23.0%), and sapelovirus A (PSV; 22.2%). To a lesser extent, other viruses present in the virome of healthy animals were porcine astroviruses (12.1%), rotaviruses (7.5%), towan virus (TV; 5.8%), and the Circovirus genus (CV; 4.6%). In moderately and severely wasting animals (*n* = 9), porcine sapovirus was the predominant virus (PSaV; 31.5%), followed by rotaviruses (23.3%), enterovirus G (EV-G; 17.1%), and porcine astroviruses (16.0%).

The last time point analyzed was 6 weeks after weaning (Figure 3E). In healthy pigs (*n* = 3), porcine picobirnavirus (PpbV; 46.5%) and porcine astroviruses (37.9%) were the dominant viruses detected, while sequences from eight other viruses were identified at a low percentage. On the other hand, porcine picobirnavirus (PpbV; 37.8%) was also a predominant virus detected in moderately and severely wasting animals (*n* = 5), together with enterovirus G (EV-G; 44.4%). In these samples, several other viruses were present at lower percentages, similar to the situation in healthy animals.

**Figure 4:**
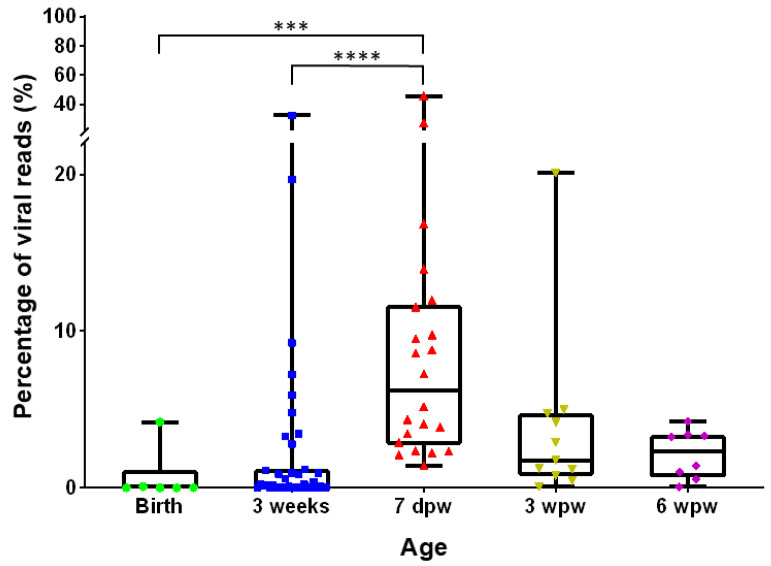
Percentage of viral reads among the total number of reads obtained in VIDISCA-NGS distributed per age group. All pairwise comparisons were performed with the Kruskal–Wallis test. The significance levels are denoted by asterisks: *** *p* ≤ 0.001, **** *p* ≤ 0.0001. If not shown, a result was not significant. The box plots show the first and third quantiles as the lower and upper hinges, the median in the center, and lowest and highest values as the whiskers. All individual samples tested are represented as colored figures per age group. Green/circle: birth; blue/square: 3 weeks; red/triangle: 7 days post weaning; yellow/inverted triangle: 3 weeks post weaning; purple/diamond: 6 weeks post weaning.

We found remarkable differences in the percentage of viral reads amongst the total of reads per sample in animals at different ages. In VIDISCA-NGS, this value is a useful indicator for the abundance of viral pathogens [12]. As shown in Figure 4, the proportion of viral reads one week after weaning (7 dpw) is significantly higher compared to the virus reads at the time of birth (Kruskal–Wallis test; *p*-value = 0.0002) and virus reads at 3 weeks of age (Kruskal–Wallis test; *p*-value < 0.0001).

Our sample collection allowed analysis of viruses in the symptom-free period early in the life of the pigs that did and those that did not develop wasting problems at 3–6 weeks post weaning. Three analysis groups were made based on the dynamics of clinical scores applied from 7 days post weaning on (Table 2). The so-called “healthy” group consists of pigs that scored 1 at all the time points (score pattern 1–1–1). The subset of pigs scored with wasting that recovered back to score 1 at 3 or 6 weeks post weaning were named the “recovered” group (score pattern 2/3–x–1 or x–2/3–1). Pigs that did not fully recover after scoring wasting, or worsened after initially being healthy, were classified as “wasting” (x–x–2/3).

**Figure 5:**
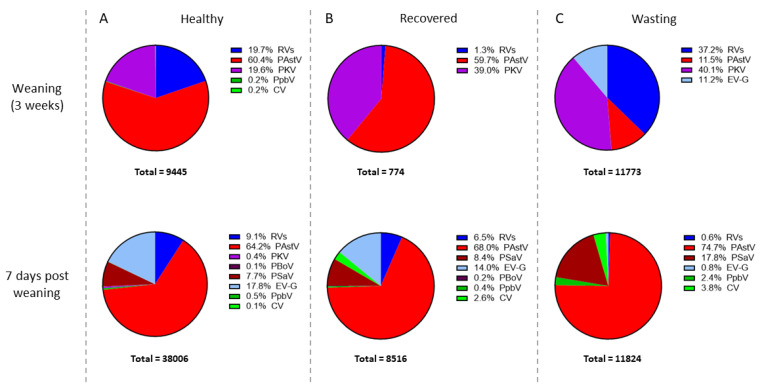
Detection of viral reads in rectal swab samples from pigs at different ages and clinical score dynamics (healthy (**A**) score pattern 1–1–1; recovered (**B**) score pattern 2/3–x–1 or x–2/3–1; wasting (**C**) score pattern x–x–2/3) using VIDISCA-NGS. The legend shows the percentage of the total number of reads allocated to each virus. RVs: rotavirus; PAstV: porcine astrovirus; CV: circovirus; PpbV: porcine picobirnavirus; PKV: porcine kobuvirus; PBoV: porcine bocavirus 3; PSaV: porcine sapovirus; EV-G: enterovirus G.

At 3 weeks of age, porcine astroviruses were the most prevalent viruses in the “healthy” (60.4%; Figure 5A, *n* = 16) and “recovered” groups (59.7%; Figure 5B, *n* = 9), with PKV (19.6% healthy; 39.0% recovered) and rotaviruses (19.7% healthy; 1.3% recovered) also being present in both groups. In contrast, the “wasting” pigs that developed a wasting phenotype showed a distinct virome composition at 3 weeks of age at that time were still healthy (Figure 5C, *n* = 14). In this wasting group, rotaviruses (37.2%) and porcine kobuvirus (PKB; 40.1%) were the predominant viruses. Interestingly, only in this wasting group was enterovirus G (EV-G; 11.2%) detected. At 7 days after weaning, when all animals were still healthy, the majority of the reads in all three analysis groups corresponded to porcine astroviruses. Remarkably, 17.83% of the reads found in the “wasting” group (Figure 5C, *n* = 5) matched with porcine sapovirus, as opposed to “recovered” (Figure 5B, *n* = 5) and “healthy” animals (Figure 5A, *n* = 12), where the second most common virus detected was enterovirus G (EV-G; 17.8% and 14.0%, respectively). This ‘tracing back’ type of analysis provides additional insight into potential virome differences between healthy and wasting pigs that were initially missed: (1) rotavirus and porcine kobuvirus are predominant viruses at 3 weeks of age in pigs that later fail to thrive; (2) enterovirus-G is only present at 3 weeks of age in pigs that later fail to thrive; (3) porcine sapovirus is a predominant virus 7 days after weaning in the wasting group compared to the healthy and recovered groups.

### 3.3. Study of the Infection Dynamics of the Different Porcine Rotavirus Species

**Figure 6:**
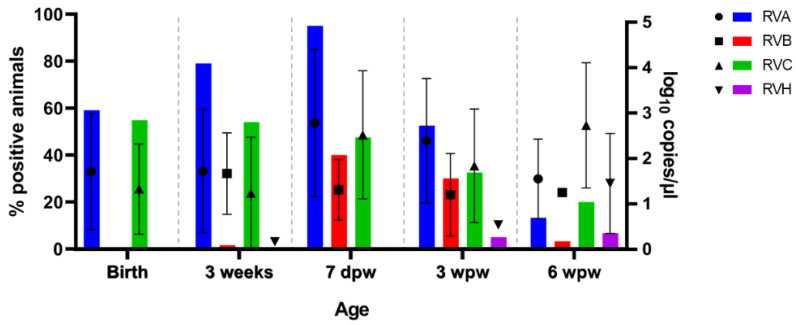
Dynamics of rotavirus infections in pigs from a farm with wasting problems at birth, 3 weeks, 7 days post weaning (7 dpw), 3 weeks post weaning (3 wpw), and 6 weeks post weaning (6 wpw). Bars show the percentage of positive animals among the total sampled animals at a certain age (left, y-axis). The viral load is expressed as log_10_ copies/µL of rectal swab extract and the mean and standard deviation are represented in the graph for each rotavirus species and pig age (right, y-axis).

We subsequently performed an in-depth analysis via quantitative PCR of two viral families that are associated with enteric disease in pigs: rotaviruses and astroviruses. The metagenomic analysis of the fecal samples showed that rotavirus species A, B, C, and H (RVA–RVC and RVH) were circulating on the farm. In order to further investigate the dynamics of infection of all species in the total group of sampled animals, specific qPCR assays were designed for each virus. Figure 6 illustrates the detection rates and viral loads of each rotavirus species at different ages.

RVA and RVC were detected at all time points and were the only ones to be detected at birth (59.1%; 55/93 and 54.8%; 51/93 prevalence, respectively). The prevalence of RVA reached its maximum at 7 days after weaning (95.0%; 38/40), also with the highest loads (2.8 ± 1.6 log_10_ copies/µL). RVC prevalence reached its maximum prevalence at 3 weeks of age (54.8%; 68/124), yet the highest viral loads were found at later time points (7 dpw: 2.5 ± 1.4 log_10_ copies/µL; 3 wpw: 1.8 ± 1.3 log_10_ copies/µL; 6 wpw: 2.7 ± 1.4 log_10_ copies/µL).

RVB infections appeared in the population at 7 days post weaning and were also detected 3 weeks after weaning (40.0%; 16/40 and 30.0%; 12/40 prevalence, respectively), yet sharply decreasing thereafter (6 wpw: 3.3%; 1/30). The presence of RVH in the farm was very low, with only few pigs positive at 3 and 6 weeks post weaning.

Figure 6 shows that rotavirus infection and viral load increase preceded the appearance of wasting symptoms in the herd starting from 7 days post weaning onwards. The differential dynamics of RV infections between healthy and wasting pigs were compared, but we saw no significant difference in the infection rates of any of the rotaviruses at any age.

We next investigated the co-infection pattern of rotaviruses in the herd. Our analysis provided evidence that almost all pigs in the population experienced an RVA infection, often in combination with RVC, especially at birth (45.2%; 42/93), weaning (50.8%; 63/124), and one week after weaning (47.5%; 19/40). Co-infection of RVB with either RVA or RVC was occasionally reported. We could not establish significant differences in the co-infection rates between healthy and wasting pigs at the different time points analyzed.

Similar to the metagenomic analysis, three analysis groups were made based on the dynamics of clinical scores since 7 days post weaning (healthy: score pattern 1–1–1; recovered: score pattern 2/3–x–1 or x–2/3–1; wasting: score pattern x–x–2/3; see Table 2). Rectal swabs were traced back up to 3 weeks of age and qPCR results were allocated to each of the groups to determine if pigs that tend to develop wasting problems had characteristic rotavirus infection patterns at earlier sampling points. Figure 5 suggests that the RV infections in the non-recovery group peaked at 3 weeks of age, earlier than in the recovered or healthy groups. The qPCR data also showed that the prevalence of RVA and RVC positive pigs in the wasting group at 3 weeks of age was higher than in the other groups, however, not statistically significant (χ2; *p*-value > 0.05; Supplementary Table S2). Furthermore, the viral loads did not show any significant differences.

### 3.4. Study of the Infection Dynamics of Distinct Porcine Astrovirus Types

**Figure 7:**
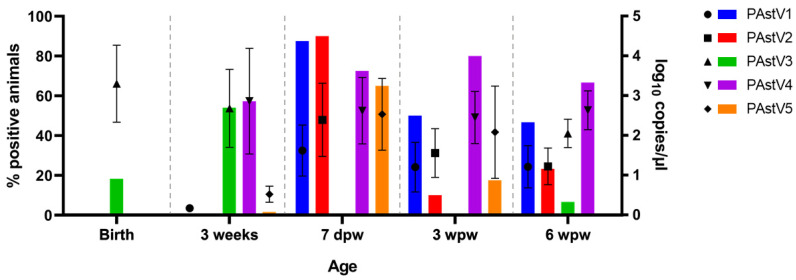
Dynamics of astrovirus infections in pigs from a farm with wasting problems at birth, 3 weeks, 7 days post weaning (7 dpw), 3 weeks post weaning (3 wpw), and 6 weeks post weaning (6 wpw). Bars show the percentage of positive animals among the total sampled animals at a certain age (left, y-axis). The viral load is expressed as log10 copies/µL of rectal swab extract, and the mean and standard deviation are represented in the graph for each rotavirus species and pig age (right, y-axis).

Two multiplex probe-based RT-PCR assays were used to analyze the infection rates and dynamics of the five different porcine astrovirus types (PAstV1-5) within the farm under study. Our analysis revealed that porcine astroviruses were highly prevalent in rectal swab samples, especially at the time of weaning and thereafter. Remarkably, 7 days after weaning all the pigs sampled were infected with one or more porcine astrovirus type (100%; 40/40). Figure 7 illustrates the detection rates and viral loads of each astrovirus type at different ages.

Among the porcine astrovirus types, PAstV4 had the highest overall prevalence in the farm. It was identified for the first time at 3 weeks of age at a rate of 57.3% (71/124) and thereafter in 72.5% (29/40) of the pigs at approximately 7 days post weaning, 80.0% (32/40) three weeks post weaning, and 66.7% (20/30) six weeks post weaning. Moreover, PAstV4 showed the highest numbers of genomic copies at each of those time points (3 weeks: 2.9 ± 1.3 log_10_ copies/µL; 7 dpw: 2.6 ± 0.8 log10 copies/µL; 3 wpw: 2.5 ± 0.7 log10 copies/µL; 6 wpw: 2.6 ± 0.5 log10 copies/µL).

At birth, PAstV3 was already present in some pigs (15.1%; 14/93) at a relatively high viral load (3.3 ± 1.0 log10 copies/µL). No other astrovirus type was detected at this age. Three weeks later, at the time of weaning, PAstV3 reached its maximum detection in the population (54.0%; 67/124). Thereafter, PAstV3 went undetected until few positives were identified 6 weeks post weaning (6.7%; 2/30).

Both PAstV1 and PAstV2 were prevalent 7 days after weaning (87.5%; 35/40 and 90.0%; 36/40 respectively). While PAstV2 detection underwent a sharp decline in the sampling times afterwards (3 wpw: 10.0%; 4/40; 6 wpw: 23.3%; 7/30), about half of the sampled population was still positive for PAstV1 at those ages (3 wpw: 50.0%; 20/40; 6 wpw: 46.7%; 14/30).

PAstV5 was overall the least prevalent virus within the population. Yet, at 7 days post weaning, 65.0% (26/40) of the sampled pigs were positive for the virus at remarkably high viral loads (2.5 ± 0.9 log10 copies/µL).

The differential dynamics of porcine astrovirus infections between healthy and wasting pigs were also studied. We found that 7 days after weaning PAstV5 infection rates in healthy pigs (81.0%; 17/21) were significantly higher than in wasting pigs (47.4%; 9/19; Fisher’s exact test, *p* = 0.046). No other significant differences in porcine astrovirus infection rates were found between both groups at any age.

Trace-back analysis was also performed on the astrovirus qPCR results up until 3 weeks of age to compare the pigs based on the dynamics of their clinical scores (healthy: score pattern 1–1–1; recovered: score pattern 2/3–x–1 or x–2/3–1; wasting: score pattern x–x–2/3; see Table 2) and determine if the wasting phenotype can be linked to characteristic astrovirus infection patterns. Although differences in prevalence between the groups could be appreciated at a few time points, no significant variations could be established (χ2; *p*-value > 0.05; Supplementary Table S3).

We next investigated if a co-infection pattern including porcine astroviruses and rotaviruses could explain the wasting phenotype. Rotavirus co-infections with PAstV3 were detected at the time of birth (11.8%; 11/93). At 3 weeks of age, co-infections with both viruses became more prevalent, with 71.0% (88/124) of the animals concurrently affected with at least one rotavirus species and one porcine astrovirus type. Among these, the most common co-infection found was RVA, RVC, and either PAstV3 (20.2%; 25/124), PAstV4 (9.7%; 12/124), or both (14.5%; 18/124). Remarkably, most of the animals at 7 days post weaning, accounting for 85.7% (18/21) of the heathy pigs and 73.7% (14/19) of the wasting pigs, were concurrently infected with at least RVA, PAstV1, and PAstV2, generally also in combination with PAstV4, PAstV5, or both. At 3 weeks post weaning, PAstV4 was the most prevalent virus in the population and, remarkably, all pigs positive for RVA were also co-infected with PAstV4. There were no significant differences in the co-infection rates between same-age pigs classified with different health scores.

## 4. Discussion

We followed a farm with recurrent problems with wasting pigs, not associated with porcine circovirus 2 (PCV2) or porcine reproductive and respiratory syndrome virus (PRRSV), with the aim of providing insight into the development of the virome over time in relation to the wasting phenotype. Starting from approximately one week after weaning, the growth of certain animals, randomly distributed across litters from both sows and gilts, appeared to cease while the weight of normal pigs quickly surpassed the weight of those clinically affected by wasting. We reported a significant reduction in body weight gain of the severely wasting pigs compared to the healthy pigs in the period between three and six weeks after weaning, as well as a reduced growth rate in the mildly to moderately affected pigs, analogous to previously reported case presentations describing marked muscle weakness and loss of body condition at that age [1,3].

Disbalances in the swine gut microbiome have been associated with reduced gut barrier function, susceptibility to pathogens, morbidities, and ultimately increased mortalities during the postweaning period [39,40]. Yet, few studies have characterized the enteric virome composition before and after weaning, and the relation between the age of the pigs and the time of sampling was mostly overlooked [25,41,42,43]. While longitudinal molecular analyses of individual viruses are fairly common in the literature, only one metagenomic study has acknowledged the correlation of the swine virome composition with age [44]. In the context of porcine failure to thrive syndrome (PFTS), Franzo et al. evaluated the virome composition of different organs in 6-week-old affected piglets and in healthy counterparts. They described a higher abundance of bocaparvovirus 2 (BoPV2), protoparvovirus 1 (PPV), and porcine circovirus 3 (PCV3) in affected pigs, but no direct correlation supporting the pathogenic role of those viruses was reported [45].

Our data show that longitudinal analysis strategies are of extra value in the analysis of complex disease with possible viral contributions. We identified 14 distinct enteric viruses within the herd. Remarkably, all the virus families studied have previously been linked to enteric diseases but are generally considered common virome components of healthy pigs [25,33,41,42,43]. Enteric viruses are already present at low levels in the farrowing period, but a clear viremia tends to occur in the week after weaning, prior to the onset of wasting problems in a subset of the pigs. During the first weeks of life, piglets receive antibodies through maternal colostrum. Loss of maternal protection towards the end of the farrowing period and after weaning could explain the increased susceptibility of the piglets to increased replication and the quick spread of diverse viruses within the herd [45].

We showed that wasting is preceded by alterations in the enteric virome with timing differences between healthy and wasting individuals. Indicative features of such ‘unbalanced’ virome are: (1) the presence of enterovirus G at 3 weeks of age; (2) rotaviruses at 3 weeks of age; and (3) porcine sapovirus one week after weaning in wasting pigs. The ubiquity of porcine sapovirus and enterovirus G at an early age correlates with the high prevalence of those viruses in the virome of sick pigs at a later age (3–6 weeks post weaning). Additionally, we showed that rotavirus infections in the non-recovery group peak at 3 weeks of age, earlier than in the recovered or healthy groups. Although our results cannot prove causality, they suggest that subclinical infections around the time of weaning may have had an impact on the development of the wasting phenotype. Moreover, we demonstrated that diagnostics sampling at one time point (at peak clinical symptoms) likely does not work for multifactorial disease, and only the in-depth study into the virome dynamics and subclinical infections in the weeks before the onset of clinical problems can give indications on the potential causality of disease.

Porcine sapoviruses are a predominant species in the virome of wasting pigs at 3 weeks post weaning, when clinical problems peak. This family of viruses is genetically diverse and widely distributed in domestic pig herds. While diarrhea has been experimentally reproduced by porcine sapovirus infection in pigs [46] and has been found in herds suffering with this problem [47], it is also widely detected in asymptomatic animals [48,49]. Histological investigation of the intestine of pigs experimentally inoculated with porcine sapovirus has shown mild to severe villous atrophy in the duodenum [46,50]. Besides the increased prevalence of porcine sapovirus in pigs that later develop a wasting phenotype, the pathological features of the virus suggest that an early infection may disrupt gut barrier function and increase susceptibility to other infectious pathogens later in life.

The presence of enterovirus G in the preweaning virome of pigs that later fail to thrive is followed by a higher prevalence of the virus in those animals at 6 weeks post weaning. The ubiquity of this virus in the worldwide pig population, generally causing asymptomatic infections, along with its genotypic diversity, may indicate that enterovirus G requires co-infections and/or co-factors to prompt clinical manifestations [51]. The dynamics of the early virome of wasting animals and the prevalence of enterovirus G and porcine sapovirus 3 weeks after weaning, when clinical symptoms peak, suggest that co-infection with both viruses may trigger the development of a wasting phenotype. In addition, enterovirus G recombinants with torovirus-like genes have been described in the literature in recent years, generally associated with diarrhea cases [52,53]. Our metagenomic results also suggest a potential recombination event between those viruses but further analysis needs to be performed to confirm this.

Porcine picobirnaviruses, highly prevalent in both healthy and wasting animals at 6 weeks post weaning, are widespread in the feces of humans and a wide variety of animals [54,55]. Although we observed significant differences in virome composition between wasting and healthy animals at 3 and 6 weeks post weaning, a causal nexus with the wasting disease may be unlikely since no clear link between infection and any disease has ever been established for this virus [56,57,58].

Our study is the first to report the dynamics and co-infection of porcine rotavirus species and porcine astrovirus genetic lineages during the first 9 weeks of the life of domestic pigs, in the context of postweaning wasting problems. No specific rotavirus genotype or porcine astrovirus type could be linked to the postweaning clinical problems in the farm, nor do the coinfections suggest any clear correlation to disease causation. Nonetheless, the high prevalence of RVA at 7 days postweaning without clinical signs of diarrhea suggests that it may be a contributing factor to virome dysbiosis. RVA and RVC, species commonly related to diarrhea in young piglets [19,59,60,61], were found to be prevalent viruses since birth until the first week after weaning. Detection of rotaviruses decreased considerably between three and six weeks after weaning, which contrasts with the report from Marthaler et al., in which RVA, RVB, and RVC were all detected at high percentages in the group of 55-day-old pigs [19].

Within the *Astroviridae* family, PAstV4 had the highest overall prevalence in the farm. Likewise, prior studies have also reported a high prevalence of PAstV4 in domestic pig farms in the US, Asia, and Europe [33,62,63,64]. It was striking, though, to find that PAstV3, which has been linked to neurological disease and polioencephalomyelitis in swine [29,65], is already prevalent in the farm before weaning. The detection of PAstV3 by in-situ hybridization in myenteric plexus neurons of piglets suggested a possible route of spread from the gastrointestinal system to the central nervous system and highlighted the endemic and pathogenic potential of this virus type within the farms [66]. Similar to our findings, previous studies have already acknowledged the diversity of viral flora in pigs and the frequent appearance of coinfections between rotaviruses, porcine astroviruses, and other intestinal pathogens, but never linked them to wasting disease [25,26,56,67].

Due to their ubiquitous presence in healthy and diseased individuals, astroviruses have gained attention as possible contributors to microbiome dysbiosis [68]. It was observed with the use of animal models that a decrease in microbial diversity in the gut occurred at the peak of astrovirus infection [45]. In addition, it has been shown that astrovirus capsid protein can disrupt the gut barrier, increasing intestinal permeability and possibly impairing sodium absorption [39,40]. In other species, astrovirus infection was associated with *E. coli* outgrowth and the development of poultry enteritis [69,70]. Astrovirus infections have also been correlated with age-related dysbiosis in bats [71]. Our study showed the widespread presence of astroviruses in pigs at all ages examined. Yet, the possible role of porcine astrovirus infections in shaping the early-life microbiome of pigs and the contribution of the microbiome as a whole to the development of the wasting phenotype should be addressed in future studies.

Although our study gave new insights into the dynamics of the virome of healthy and wasting pigs, we were unable to confirm causal links with clinical wasting. The heterogeneity of virus infections and the immune responses evoked could influence many of the complex inflammatory diseases of yet unknown causation [72,73]. Evidence suggests that continuous exposure to viral infections stimulates the immune system and, potentially, effects resistance to other infections, or contributes to the development of certain diseases, such as type 1 diabetes, IBD, and asthma [74]. Research into subclinical viral coinfections with enteric viruses and their role in the immunomodulation of multifactorial complex diseases is still limited. A recent publication from Dallari et al. studied the evoked responses to several enteric viruses in the absence of disease manifestations to understand the immune spaces occupied by the enteric virome. Their results showed that each virus is associated with a unique immune profile and suggested that long-term immunomodulation is possible with only transient infection early in life [75,76]. From our data it appears that the timing of the viremic peak rather than the presence of the virus influences susceptibility to wasting.

The appearance of wasting disease in pig herds historically free of PCV2 and other known causative pathogens in the last decade indicated a potential distinct but yet unknown aetiology of the disease [3]. Several studies have suggested a non-infectious origin of the disease [77,78]. Inoculation of snatch-farrowed porcine colostrum-deprived pigs with pooled organ homogenates from affected pigs failed to experimentally reproduce the characteristic disease symptomatology [79]. Contrary to those who considered a direct viral causation of disease, we hypothesized that the characteristic symptomatology known as failure to thrive or wasting not related to PCV2 infection may be triggered by complex interactions within the pig virome and/or microbiome already as early as the pre-weaning stage and leads, in some cases, to wasting disease once the pigs are weaned. The current study provides valuable insight into the formation of the enteric virome and the potential for viral genera to affect pig herds worldwide that are as yet unknown and are not associated with clear characteristic pathogeneses. It will be difficult, however, to establish an animal model to confirm such interactions, especially because we see that within litters both future healthy and wasting piglets reside in the same environment, suggesting that there may be a crucial contribution of maternal immunity towards controlling the replication of enteric viral pathogens.

In conclusion, our study is the first to evaluate a longitudinal virome composition in the context of a farm affected with postweaning wasting problems. We demonstrated the existence of a rich enteric virome in both symptomatic and asymptomatic animals, which includes viruses previously linked to enteric or neurological disorders and which is highly dependent on age and clinical status. We showed that even at the preweaning stage, enteric virus infections may have a determining role in the future development of intestinal barrier dysfunctions and subsequent wasting [6]. We hypothesize that this impairment affects nutrient resorption and growth and thus influences the onset of failure to thrive pathogenesis, reflected later in life in a different enteric virome and presumably also microbiome.

## Figures and Tables

**Table 1:** Number of samples selected for metagenomic analysis from each age group and clinical condition.

Clinical Condition	Birth	3 Weeks	7 dpw ^a^	3 wpw	6 wpw
Healthy	6	39	14	2	3
Wasting	n/a	n/a	8	9	5

^a^ dpw: days post weaning; wpw: weeks post weaning.

**Table 2:** Dynamics of clinical scores during the postweaning period.

Clinical Symptoms 7 dpw ^a^	Clinical Symptoms 3 wpw	Clinical Symptoms 6 wpw	Number of Animals (%)
Score 1 ^b^	Score 1	Score 1	230/377 (61.0%)
Score 2	Score 1	Score 1	18/377 (4.8%)
Score 1/2	Score 2: *n* = 32 Score 3: *n* = 7	Score 1	39/377 (10.3%)
Score 1/2	Score 1: *n* = 36 Score 2: *n* = 32 Score 3: *n* = 13	Score 2	81/377 (21.5%)
Score 1/2	Score 1: *n* = 1 Score 2: *n* = 4 Score 3: *n* = 4	Score 3	9/377 (2.4%)

^a^: dpw: days post weaning; wpw: weeks post weaning; ^b^ Score 1—healthy appearance; Score 2—observable lower weight combined with some loss of typical finisher-type musculature, mild to moderate wasting; Score 3—severely reduced weight and extensive loss of musculature, severe wasting, dark green: healthy; light green: recovered; orange: wasting.

## Supplementary Materials

The following are available online at https://www.mdpi.com/article/10.3390/v13122538/s1, Table S1: Primer and probe sequences and stock concentrations for rotaviruses and porcine astroviruses qPCR assays; Table S2: Prevalence and viral loads in rectal swab samples of RVA, RVB, RVC and RVH from pigs at different ages and clinical score dynamics; Table S3: Prevalence and viral loads in rectal swab samples of PAstV1, PAstV2, PAstV3, PAstV4 and PAstV5 from pigs at different ages and clinical score dynamics.
