# Supplementary material for: Dynamics of the Enteric Virome in a Swine Herd Affected by Non-PCV2/PRRSV Postweaning Wasting Syndrome
Source: Viruses. 2021 Dec 17;13(12):2538. doi: 10.3390/v13122538 (PMC8705478; doi:10.3390/v13122538)
Supplement: Supplementary file 1 [file viruses-13-02538-s001.zip › viruses-1463546-supplementary.pdf]

viruses

MDPI

Supplementary Materials

Dynamics of the Enteric Virome in a Swine Herd Affected by Non-PCV2/PRRSV Postweaning Wasting Syndrome

Table S1. Primer and probe sequences and stock concentrations for rotaviruses and porcine astroviruses qPCR assays.

Primer/Probe	Concentration	Sequence
RVA forward primer	40 µM	5′-GCT AGG GAY AAA ATT GTT GAA GGT A-3′
RVA reverse primer	40 µM	5′-ATT GGC AAA TTT CCT ATT CCT CC-3′
RVA probe 1	5 µM	5′-FAM-ATG AAT GGA AAT GAY TTT CAA AC-MGB-3′
RVA probe 2	5 µM	5′-FAM-ATG AAT GGA AAT AAT TTT CAA AC-MGB-3′
RVC forward primer	40 µM	5′-ATG TAG CAT GAT TCA CGA ATG GG-3′
RVC reverse primer	40 µM	5′-ACA TTT CAT CCT CCT GGG GAT C-3′
RVC probe	5 µM	5′-VIC-GCG TAG GGG CAA ATG CGC ATG A-TAMRA-3′
RVB forward primer	10 µM	5′-AGT CGG ACC TTT ACA GAC GA-3′
RVB reverse primer	10 µM	5′-GCA GGC GTC ATA TGG AGA AG-3′
RVH forward primer	10 µM	5′- GGA CTA AAG AAG GAG CTG CC-3′
RVH reverse primer	10 µM	5′- TCA GTC CTG TCC ATC ACC AT-3′
PAstV forward primer	10 µM	5′-CTS YAT GGG AAA CTC CTK WGY-3′
PAstV reverse primer	10 µM	5′-YTT TGG TCC KCC CCY CCA AA-3′
PAstV1 probe	10 µM	5′-FAM-ATG GAC GAG /ZEN/ GAA CAT CCC TTC AAA TGC T-3IABkFQ-3′
PAstV2 probe	10 µM	5′-SUN-CCG GGC GCA /ZEN/ CCT TGC CTA AAA TC-3IABkFQ-3′
PAstV3 probe	10 µM	5′-FAM-TCC TTG GCC /ZEN/ ATA ACC TCC CTG ATG ACA-3IABkFQ-3′
PAstV4 probe	10 µM	5′-SUN-AAA TGT TTG /ZEN/ GCT GAA ACA GCG AGG CAC-3IABkFQ-3′
PAstV5 probe	10 µM	5′-ROX-CTC GTG TTG GCT CTG ATC TGC CAG TCT T-3IAbRQSp-3′

Table S2. Prevalence and viral loads in rectal swab samples of RVA, RVB, RVC and RVH from pigs at different ages and clinical score dynamics.

Age	Porcine Rotavirus Specie	Prevalence and viral load[a]	Healthy[b]	Recovered	Wasting
3 weeks	RVA	*n*	61/76	11/19	26/29
		log10 cop/ µL	1.79 ± 1.32	1.43 ± 0.91	1.67 ± 1.62
	RVB	*n*	0/76	1/19	0/29
		log10 cop/ µL	n/a	2.30	n/a
	RVC	*n*	37/76	10/19	20/29
		log10 cop/ µL	1.08 ± 1.08	1.35 ± 0.77	1.52 ± 1.62
	RVH	*n*	0/76	1/19	0/29
		log10 cop/ µL	n/a	0.17	n/a
7 dpw	RVA	*n*	17/17	12/13	9/10
		log10 cop/ µL	2.62 ± 1.45	2.71 ± 1.88	3.20 ± 1.70
	RVB	n	8/17	3/13	5/10
		log10 cop/ µL	1.18 ± 0.39	1.67 ± 1.30	1.30 ± 0.65
	RVC	n	9/17	5/13	5/10
		log10 cop/ µL	2.40 ± 1.28	3.57 ± 1.30	1.71 ± 1.34
	RVH	n	0/17	0/13	0/10
		log10 cop/ µL	n/a	n/a	n/a
3 wpw	RVA	n	13/20	2/5	6/15
		log10 cop/ µL	2.49 ± 1.41	2.13 ± 2.63	2.25 ± 1.15
	RVB	n	6/20	1/5	5/15
		log10 cop/ µL	1.64 ± 1.12	0.49	0.81 ± 0.32
	RVC	n	7/20	2/5	4/15
		log10 cop/ µL	1.78 ± 1.20	1.97 ± 0.61	1.89 ± 1.78
	RVH	n	1/20	0/5	1/15
		log10 cop/ µL	0.49	n/a	0.59
6 wpw	RVA	n	1/7	0/4	3/19
		log10 cop/ µL	1.75	n/a	1.49 ± 1.06
	RVB	n	1/7	0/4	0/19
		log10 cop/ µL	1.25	n/a	n/a
	RVC	n	2/7	0/4	4/19
		log10 cop/ µL	3.54 ± 2.53	n/a	2.33 ± 0.63
	RVH	n	0/0	0/0	2/19
		log10 cop/ µL	n/a	n/a	1.45 ± 1.10

[a] Prevalence is expressed as the number of positive pigs/number of pigs in the analysis group. Viral loads in rectal swab samples are expressed as log10 copies/µL of rectal swab extract. [b] Clinical score dynamics: healthy: score patter 1-1-1; recovered: score pattern 2/3-x-1 or x-2/3-1; wasting: score pattern x-x-2/3.

Table S3. Prevalence and viral loads in rectal swab samples of PAstV1, PAstV2, PAstV3, PAstV4 and PAstV5 from pigs at different ages and clinical score dynamics.

Age	Porcine Astrovirus Lineages	Prevalence and viral load [a]	Healthy [b]	Recovered	Wasting
3 weeks	PAstV1	n	0/76	1/19	0/29
		log10 cop/ µL	n/a	0.17	n/a
	PAstV2	n	0/76	0/19	0/29
		log10 cop/µL	n/a	n/a	n/a
	PAstV3	n	36/76	12/19	19/29
		log10 cop/µL	2.59 ± 0.82	2.88 ± 1.03	2.67 ± 1.23
	PAstV4	n	46/76	9/19	16/29
		log10 cop/µL	2.88± 1.43	2.81 ± 1.03	2.83 ± 1.26
	PAstV5	n	0/76	2/19	0/29
		log10 cop/µL	n/a	0.52 ± 0.20	n/a
7 dpw	PAstV1	n	16/17	11/13	8/10
		log10 cop/µL	1.60 ± 0.54	1.70 ± 0.80	1.56 ± 0.66
	PAstV2	n	15/17	12/13	9/10
		log10 cop/µL	2.62 ± 0.78	2.18 ± 0.98	2.26 ± 1.06
	PAstV3	n	0/17	0/13	0/10
		log10 cop/µL	n/a	n/a	n/a
	PAstV4	n	14/17	10/13	5/10
		log10 cop/µL	2.57 ± 0.82	2.81 ± 0.67	2.36 ± 1.24
	PAstV5	n	15/17	5/13	6/10
		log10 cop/µL	2.40 ± 1.01	2.59 ± 0.61	2.82 ± 0.87
3 wpw	PAstV1	n	8/20	2/5	10/15
		log10 cop/µL	1.43 ± 0.74	1.12 ± 0.20	1.04 ± 0.55
	PAstV2	n	1/20	1/5	2/15
		log10 cop/µL	0.72	2.00	1.76 ± 0.39
	PAstV3	n	0/20	0/5	0/15
		log10 cop/µL	n/a	n/a	n/a
	PAstV4	n	16/20	5/5	11/15
		log10 cop/µL	2.51 ± 0.77	2.43 ± 0.45	2.37 ± 0.60
	PAstV5	n	2/20	1/5	4/15
		log10 cop/µL	1.67 ± 0.69	1.28	2.49 ± 1.40
6 wpw	PAstV1	n	4/7	3/4	7/19
		log10 cop/µL	1.26 ± 0.52	1.06 ± 0.37	1.25 ± 0.64
	PAstV2	n	1/7	2/4	4/19
		log10 cop/µL	1.31	1.27 ± 0.92	1.18 ± 0.37
	PAstV3	n	0/7	1/4	1/19
		log10 cop/µL	n/a	2.30	1.80
	PAstV4	n	4/7	1/4	15/19
		log10 cop/µL	2.95 ± 0.66	2.85	2.53 ± 0.43
	PAstV5	n	0/7	0/4	0/19
		log10 cop/µL	n/a	n/a	n/a

[a] Prevalence is expressed as the number of positive pigs/number of pigs in the analysis group. Viral loads in rectal swab samples are expressed as log10 copies/µL of rectal swab extract. [b] Clinical score dynamics: healthy: score patter 1-1-1; recovered: score pattern 2/3-x-1 or x-2/3-1; wasting: score pattern x-x-2/3.
